# Comparative efficacy and safety of immune checkpoint inhibitor-related therapies for advanced melanoma: a Bayesian network analysis

**DOI:** 10.18632/oncotarget.18906

**Published:** 2017-07-01

**Authors:** Xin Li, Junpeng Wang, Yun Yao, Lei Yang, Zhiqin Li, Cheng Yu, Peiyan Zhao, Yongli Yu, Liying Wang

**Affiliations:** ^1^ Department of Molecular Biology, College of Basic Medical Sciences, Norman Bethune Health Science Center, Jilin University, Changchun 130021, China; ^2^ Department of Urology, Henan Provincial People's Hospital, Zhengzhou 450003, China; ^3^ Department of Immunology, College of Basic Medical Sciences, Norman Bethune Health Science Center, Jilin University, Changchun 130021, China; ^4^ Department of Epidemiology and Biostatistics, School of Public Health, Jilin University, Changchun 130021, China

**Keywords:** immune checkpoint inhibitor, therapy, efficacy, safety, advanced melanoma

## Abstract

**Objectives:**

We aimed to compare and rank the effects of 9 immune checkpoint inhibitor-related therapies for treating advanced melanoma.

**Methods:**

We searched Pubmed, Cochrane databases, Web of Science, and ClinicalTrials.gov for randomized controlled trials of the immune checkpoint inhibitor-related treatments for advanced melanoma. Analysis was done on a Bayesian framework.

**Results:**

Twelve trials including 5413 patients were identified. Ipilimumab plus nivolumab, nivolumab, and pembrolizumab were significantly more efficacious for progression-free survival (PFS) than ipilimumab (hazard ratio [HR], 0.38, 0.50, and 0.58, respectively), ipilimumab plus chemotherapy (0.45, 0.60, and 0.70, respectively), or ipilimumab plus sargramostim (0.44, 0.57, and 0.67, respectively). Ipilimumab plus gp100 was significantly less efficacious for PFS than the remaining eight immune checkpoint inhibitor-related strategies. Pembrolizumab was significantly more efficacious than ipilimumab and ipilimumab plus gp100 (HR, 0.66, and 0.64, respectively) in improving overall survival (OS). Nivolumab significantly improved OS over tremelimumab (HR, 0.48). Ipilimumab plus sargramostim was ranked the second most effective strategy in terms of OS and well tolerated. Nivolumab and pembrolizumab showed the best profile of acceptability, with significantly less high-grade adverse events than ipilimumab (odds ratio [OR], 0.49 and 0.50, respectively), tremelimumab (0.21 and 0.21, respectively), ipilimumab plus chemotherapy (0.13 and 0.13, respectively), or ipilimumab plus nivolumab (0.15 and 0.15, respectively).

**Conclusions:**

Nivolumab, pembrolizumab and ipilimumab plus sargramostim might be optimum treatments for advanced melanoma because they have the most favorable balance between benefits and acceptability. Ipilimumab plus nivolumab is the most effective in prolonging PFS, but is far more toxic than nivolumab and pembrolizumab.

## INTRODUCTION

The incidence of melanoma with high risk of causing death has increased over the past 40 years [1]. Although only constituting 4% of skin cancers, melanoma is responsible for approximately 80% of skin-cancer related deaths [2]. The death was mainly caused by metastases to the lymphatic system and other organs in the patients with advanced melanoma [3, 4]. Undergone conventional chemotherapies, the median overall survival (OS) time of the patients with stage III or IV melanoma was only 6–9 months and the 5-year survival rate was under 5% [5].

In recent years, three humanized monoclonal antibodies, ipilimumab, nivolumab and pembrolizumab, have been licensed for the treatment of advanced melanoma. Ipilimumab is the first licensed immune checkpoint inhibitor (ICI) targeting cytotoxic T lymphocyte-associated antigen-4 (CTLA-4). Nivolumab and pembrolizumab are another type of ICI targeting programmed death 1 (PD-1). Clinically, these three antibodies significantly prolonged the survival of patients with advanced melanoma [6–8]. In addition, tremelimumab, another anti-CTLA-4 monoclonal antibody was tested in phase III clinical trials [9, 10]. Noticeably, the responses to single-agent anti-PD-1 or anti-CTLA-4 therapy are more often partial than complete in majority of the patients [11]. To improve their efficacy, the licensed antibodies have been evaluated in varied combinations for the treatment of advanced melanoma in clinical trials.

In a phase III trial, although accompanied by severe adverse events (AEs), ipilimumab plus nivolumab significantly increased overall response rate (ORR) and prolonged progression-free survival (PFS) compared to ipilimumab or nivolumab treatment alone (ORR: 57.6% vs 19% vs 43.7%; PFS: 11.5 months vs 2.9 months vs 6.9 months) in patients with advanced melanoma [12]. In addition to ICI, ipilimumab has also been administrated with dacarbazine [13], the glycoprotein 100 (gp100) peptide vaccine [9], granulocyte-macrophage colony-stimulating factor (GM-CSF)-secreting tumor vaccine [14] or budesonide [15] for treating the patients with advanced melanoma. Although progress has been made, controversies remain on selecting the optimal regimen for the treatment of advanced melanoma. Thus, it is necessary to compare the efficacy and safety of the ICI-related therapies for providing guidance for their optimal use. However, the lack of head-to-head trials and varied efficacy measures among different trials limit the direct comparation of the ICI-related therapies using traditional meta-analysis methods.

In this study, we performed a network meta-analysis on all available randomized evidences to comprehensively compare and rank ICI-related therapies for the treatment of advanced melanoma. The therapies were compared for their efficacies and safeties using both direct comparisons of treatments within randomized controlled trials (RCTs) and indirect comparisons between trials based on a common comparator [16, 17]. The analysis that may provide guidance to opmize the ICI-related therapies for advanced melanoma.

## RESULTS

### Search results and study characteristics

**Figure 1:**
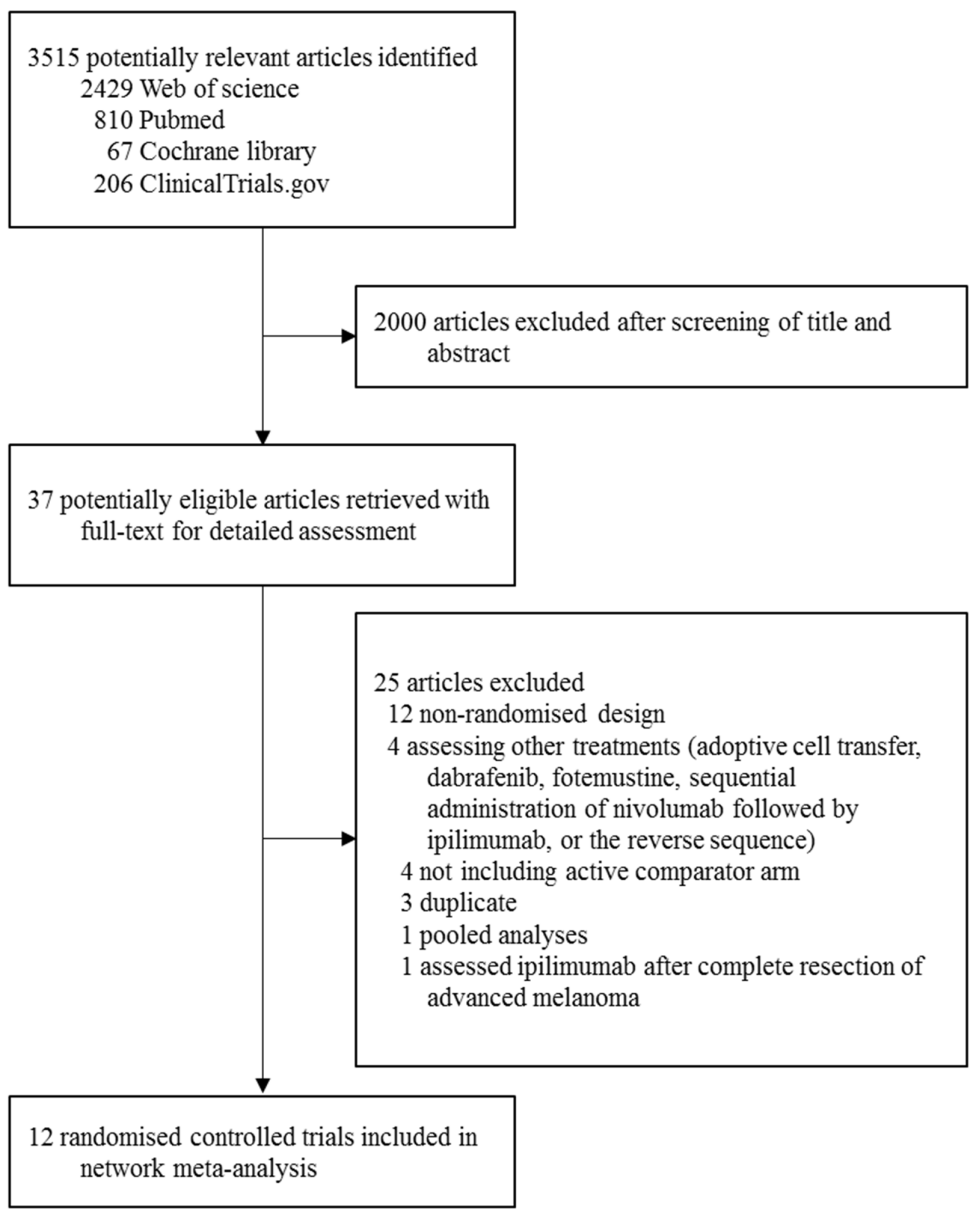
Literature search and selection

**Figure 2:**
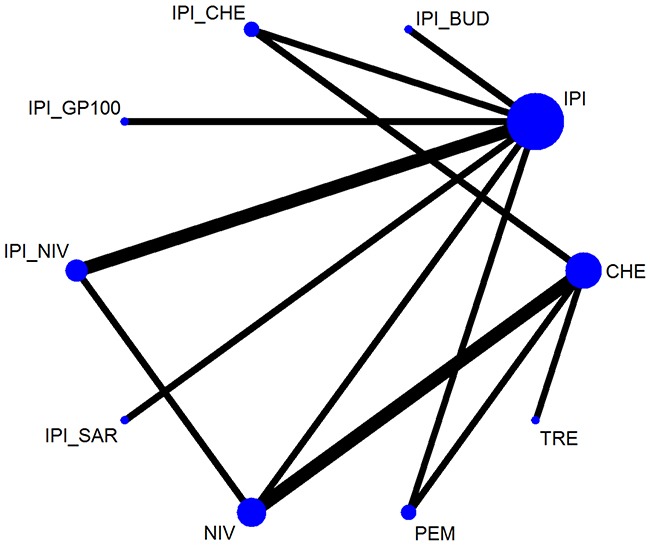
Network of the comparisons for the Bayesian network meta-analysis CHE = chemotherapy. IPI = ipilimumab. TRE = tremelimumab. NIV = nivolumab. PEM = pembrolizumab. IPI_CHE = ipilimumab plus chemotherapy. IPI_NIV = ipilimumab followed by nivolumab. IPI_GP100 = ipilimumab plus glycoprotein 100. IPI_BUD = ipilimumab plus budesonide. IPI_SAR = ipilimumab plus sargramostim.

The literature search yielded 3515 potentially eligible reports, of which 2470 were excluded after screening titles and abstracts (Figure 1). The full text of 45 remaining articles were analyzed, and finally 12 studies were included (Table 1), involving 5413 patients randomly assigned to one of the ten treatment strategies: chemotherapy, ipilimumab, tremelimumab, nivolumab, pembrolizumab, ipilimumab plus chemotherapy, ipilimumab plus nivolumab, ipilimumab plus gp100, ipilimumab plus budesonide, and ipilimumab plus sargramostim (Figure 2). The median number of participants was 547 (range, 72-945). The median follow-up period reported across all studies ranged from 6.8 to 36.6 months. Six studies were two-arm trials; and six were three-arm trials with three different comparisons.

**Table 1:** Studies included in the multiple-treatments meta-analysis

Study	Number of patients	Age (years)median (range)	Sex (% female)	Follow-up (months)median (IQR)	Median PFS in months (95% CI)	Hazard ratio (95% CI)
Weber et al. (2015) [26]						
Nivolumab 3 mg/kg every 2 weeks	272	59 (23-88)	35	8.4 (7.0-9.8)	NA	0.82 (0.32-2.05)
Investigator choice chemotherapy	133	62 (29-85)	48	8.4 (7.0-9.8)	NA	1 (Ref)
Ribas et al. (2015) [27]						
Pembrolizumab 2 mg/kg	180	62 (15-87)	42	10 (8-12)	2.9 (2.8-3.8)	0·57 (0.45-0.73)
Pembrolizumab 10 mg/kg	181	60 (27-89)	40	10 (8-12)	2.9 (2.8-4.7)	0.50 (0.39-0.64)
Investigator choice chemotherapy	179	63 (27-87)	36	10 (8-12)	2.7 (2.3-2.8)	1 (Ref)
Larkin et al. (2015) [12]						
Nivolumab (3 mg/kg every 2 weeks) followed by placebo	316	59 (25-90)	36.1	(12.2-12.5)	6.9 (4.3-9.5)	0.57 (0.43-0.76)
Ipilimumab (3 mg/kg every 3 weeks) followed by nivolumab (1 mg/kg every 2 weeks)	314	59 (18-88)	34.4	(12.2-12.5)	11.5 (8.9-16.7)	0.42 (0.31-0.57)
Ipilimumab (3 mg/kg every 3weeks) followed by placebo	315	61 (18-89)	35.9	(12.2-12.5)	2.9(2.8-3.4)	1 (Ref)
Postow et al. (2015) [28]						
Ipilimumab (3 mg/kg every 3weeks) followed by nivolumab (1 mg/kg)	95	64 (27-87)	34	>11	Not reached	0.4 (0.23-0.68)
Ipilimumab (3 mg/kg every 3weeks) followed by placebo	47	67 (31-80)	32	>11	4.4 (2.8-5.7)	1 (Ref)
Robert et al. (2015) [29]						
Pembrolizumab 10 mg/kg every 2 weeks	279	61 (18-89)	42.3	7.9 (6.1-11.5)	5.5 (3.4-6.9)	0.58 (0.46-0.72)
Pembrolizumab 10 mg/kg every 3 weeks	277	63 (22-89)	37.2	7.9 (6.1-11.5)	4.1 (2.9-6.9)	0.58 (0.47-0.72)
Ipilimumab (3 mg/kg) every 3 weeks	278	62 (18-88)	41.7	7.9 (6.1-11.5)	2.8 (2.9-2.8)	1 (Ref)
Robert et al. (2015) [30]						
Nivolumab (3 mg/kg) every3 weeks	210	64 (18-86)	42.4	8.9	5.1 (3.5-108)	0.43 (0.34-0.56)
Dacarbazine	208	66 (26-87)	39.9	6.8	2.2 (2.1-2.4)	1 (Ref)
Hodi et al. (2014) [14]						
Ipilimumab (10 mg/kg) every 3 weeks combined with sargramostim	123	61 (25-86)	30.9	13.3 (0.03-19.9) ^a^	3.1 (2.9-4.6)	0.87 (0.64-1.18)
Ipilimumab (10 mg/kg) every 3 weeks	122	64 (21-89)	36.1	13.3 (0.03-19.9) ^a^	3.1 (2.9-4.0)	1 (Ref)
Ribas et al. (2013) [10]						
Tremelimumab (15 mg/kg every 90 days)	328	57 (22-90)	42	NA	NA	0.55 (0.39-0.76)
Investigator choice chemotherapy	327	56 (22-90)	44	NA	NA	1 (Ref)
Robert et al. (2011) [31]						
Ipilimumab (10 mg/kg every 3 weeks) combined with dacarbazine	250	57.5 ^c^	39.2	36.6	NA	0.76 (0.63-0.93)
Dacarbazine plus placebo	252	56.4 ^c^	40.9	36.6	NA	1 (Ref)
Hodi et al. (2010) [9]						
Ipilimumab (3 mg/kg every 3 weeks) plus glycoprotein 100	403	55.6 ^c^	38.7	21	2.76 (2.73-2.79)	0.81 (0.74-0.92)
Ipilimumab (3 mg/kg every 3 weeks)	137	56.8 ^c^	40.9	27.8	2.86 (2.76-3.02)	0.64 (0.56-0.78)
Glycoprotein 100	136	57.4 ^c^	46.3	17.2	2.76 (2.73-2.83)	1 (Ref)
Hersh et al. (2011) [13]						
Ipilimumab (3 mg/kg every 4 weeks) plus dacarbazine	35	60 (27-82)	25.7	20.9	NA	NA
Ipilimumab (3 mg/kg every 4 weeks)	37	66 (25-82)	43.2	16.4	NA	NA
Weber et al. (2009) [15]						
Ipilimumab (10 mg/kg every3 weeks) plus budesonide	58	58 (30-82)	26	12.6 (5.6-22.1) ^b^	NA	NA
Ipilimumab (10 mg/kg every3 weeks) plus placebo	57	61 (26-86)	33	16.3 (9.1-21.4)^b^	NA	NA

PFS = progression-free survival. IQR = interquartile range. CI = confidence interval. NA = data not available. Ref = reference group (hence hazard ratio set to 1). a range. b 95% CI. c mean.

In the main text, the network meta-analysis results were reported based on fixed-effects models because they showed better goodness of fit than random-effects models. The results of random-effects models are showed in Supplementary Materials (Supplementary Table 3–6).

### Progression-free survival

**Figure 3:**
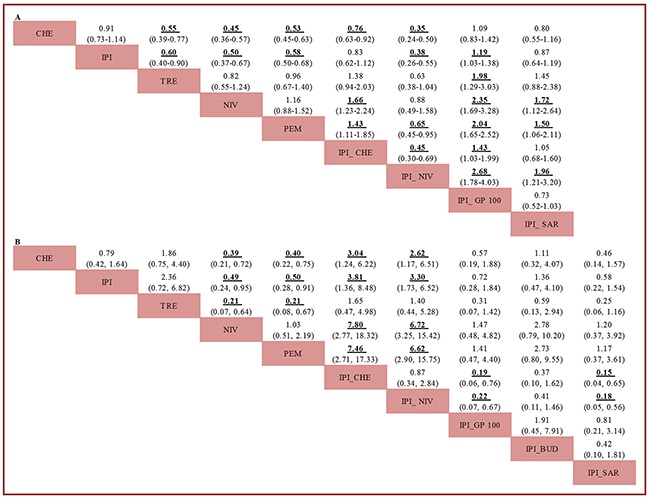
Pooled hazard ratios for progression-free survival **(A)** and pooled odds ratios for high-grade adverse events **(B)**. The column treatment is compared with the row treatment. For progression-free survival, HRs lower than 1 favor the column-defining treatment. For high-grade adverse events, ORs lower than 1 favor the column-defining treatment. Numbers in parentheses indicate 95% credible intervals. Significant results are in bold and underscored. CHE = chemotherapy. IPI = ipilimumab. TRE = tremelimumab. NIV = nivolumab. PEM = pembrolizumab. IPI_CHE = ipilimumab plus chemotherapy. IPI_NIV = ipilimumab followed by nivolumab. IPI_GP100 = ipilimumab plus glycoprotein 100. IPI_BUD = ipilimumab plus budesonide. IPI_SAR = ipilimumab plus sargramostim.

A total of ten trials involving 5226 patients provided adequate information on PFS and were included for network meta-analysis [9, 10, 12, 14, 26–31]. We summarized the results of multiple treatments meta-analysis for PFS in Figure 3A. Compared with chemotherapy, tremelimumab, nivolumab, pembrolizumab, ipilimumab plus chemotherapy and ipilimumab plus nivolumab were associated with a lower rate of progression. Nivolumab, pembrolizumab, and ipilimumab plus nivolumab were more effective than ipilimumab, ipilimumab plus chemotherapy, and ipilimumab plus sargramostim. Ipilimumab plus nivolumab was shown to be the most effective treatment, with a non-significant hazard ratio (HR) of 0.63 (0.38-1.04) versus tremelimumab and 0.88 (0.49-1.58) versus nivolumab, and significant differences for the remaining strategies, with HRs ranging from 0.32 to 0.65 favoring ipilimumab plus nivolumab. Additionally, ipilimumab plus gp100 was the least effective treatment among all treatment strategies evaluated in this study except chemotherapy.

### Overall survival

A total of eight studies, with 3381 enrolled patients, contributed to the analysis of OS [9, 10, 13–15, 29–31]. The results demonstrated that nivolumab, pembrolizumab, and ipilimumab plus chemotherapy were associated with significantly higher improvement in OS than chemotherapy (Supplementary Figure 2). Pembrolizumab was more effective than ipilimumab (HR, 0.66; 95% CI, 0.54-0.80) and ipilimumab plus gp100 (HR, 0.64; 95% CI, 0.47-0.86) in improving OS. In addition, nivolumab significantly improved OS over tremelimumab (HR, 0.48; 95% CI, 0.27-0.83). Stepwise comparison of all other treatment strategies did not find significant differences in overall survival.

### Adverse events

All the 12 trials contributed to the analysis of overall and high-grade drug-related AEs [9, 10, 12–15, 26–31]. The results of comparisons of AEs caused by the 10 treatment strategies are available in Figure 3B and Supplementary Figure 2. Nivolumab and pembrolizumab were associated with the lower rate of high-grade AEs than chemotherapy, ipilimumab and tremelimumab. In comparison with ipilimumab plus chemotherapy and ipilimumab plus nivolumab, all treatment regimens except ipilimumab plus budesonide were associated with less high-grade AEs.

### Ranking

**Figure 4:**
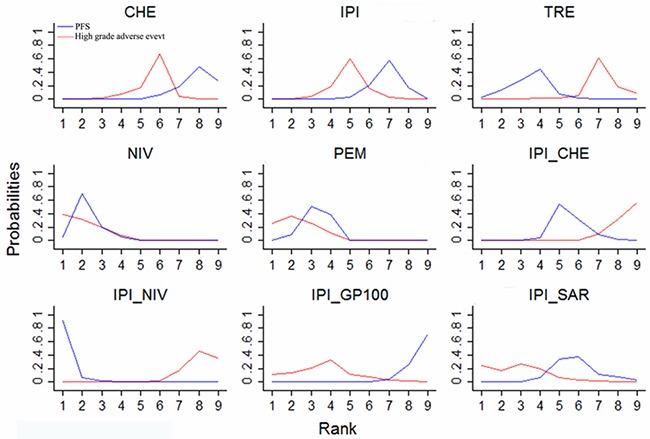
Ranking of treatments in terms of progression-free survival benefit (blue line) and high-grade adverse events (red line) Ranking indicates the probability to be the best treatment, the second best, the third best, and so on, among the 9 immune checkpoint inhibitor-related treatments. CHE = chemotherapy. IPI = ipilimumab. TRE = tremelimumab. NIV = nivolumab. PEM = pembrolizumab. IPI_CHE = ipilimumab plus chemotherapy. IPI_NIV = ipilimumab followed by nivolumab. IPI_GP100 = ipilimumab plus glycoprotein 100. IPI_SAR = ipilimumab plus sargramostim.

To select optimal therapy for advanced melanoma, we ranked the ten therapies in terms of PFS (Figure 4), OS (Supplementary Figure 3), overall adverse events (Supplementary Figure 4) or high-grade adverse events (Figure 4). Ipilimumab plus nivolumab, nivolumab and pembrolizumab were most likely to be the three best treatments according to PFS. Nivolumab, pembrolizumab, and ipilimumab plus sargramostim were the best tolerated therapies among all analyzed treatment strategies. Overall, nivolumab and pembrolizumab had similar absolute effects and ranking, and were more favorable according to the balance between therapy benefit and risk.

### Network assumptions, sensitivity analysis, publication bias and risk of bias

**Figure 5:**
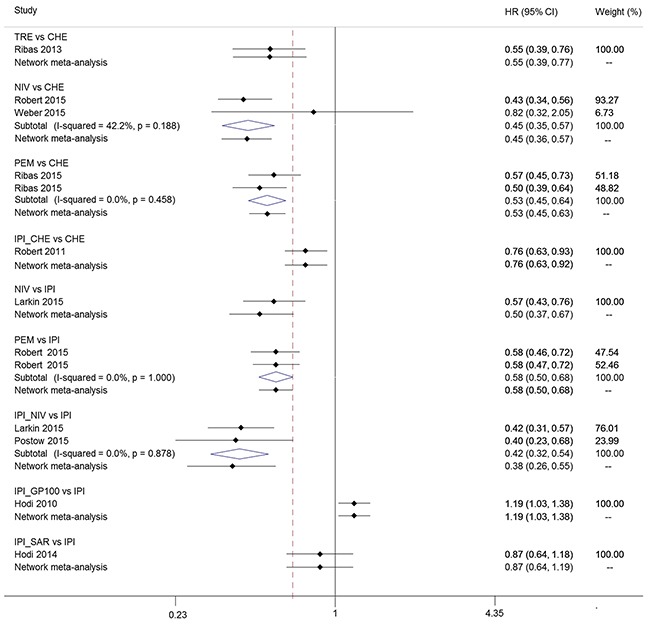
Pooled hazard ratios for progression-free survival by Bayesian network-analysis and traditional meta-analysis HR = hazard ratio. CI=confidence interval for traditional meta-analysis and credible interval for Bayesian network meta-analysis. CHE = chemotherapy. IPI = ipilimumab. TRE = tremelimumab. NIV = nivolumab. PEM = pembrolizumab. IPI_CHE = ipilimumab plus chemotherapy. IPI_NIV = ipilimumab followed by nivolumab. IPI_GP100 = ipilimumab plus glycoprotein 100. IPI_SAR = ipilimumab plus sargramostim.

**Figure 6:**
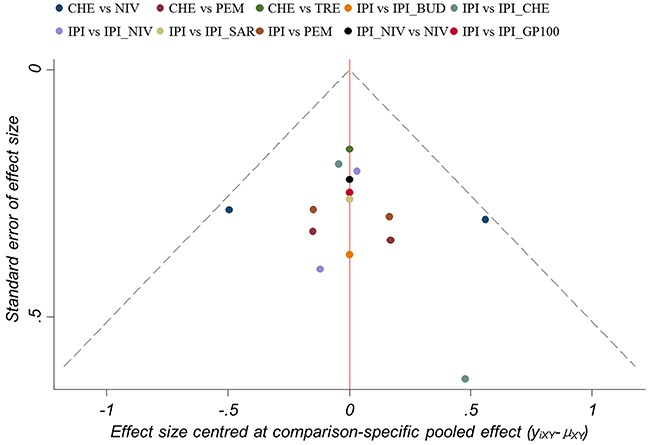
Funnel plot of randomized controlled trials included in the meta-analysis for hazard ratios of progression-free survival CHE = chemotherapy. IPI = ipilimumab. TRE = tremelimumab. NIV = nivolumab. PEM = pembrolizumab. IPI_CHE = ipilimumab plus chemotherapy. IPI_NIV = ipilimumab followed by nivolumab. IPI_GP100 = ipilimumab plus glycoprotein 100. IPI_BUD = ipilimumab plus budesonide. IPI_SAR = ipilimumab plus sargramostim.

Consistent results between direct and indirect estimates were noted for any outcome (Figure 5, Supplementary Figure 5, Supplementary Table 1 and Supplementary Table 2). The results from the sensitivity analyses were consistent with the primary analysis (Supplementary Table 7). The funnel plot (Figure 6) for PFS was largely symmetric, demonstrating no apparent small-study effects and publication bias. The methodological quality was satisfactory in the included trials (Supplementary Figure 1). There was no definite high risk of bias for random sequence generation, allocation concealment, incomplete outcome data, and selective reporting of outcomes. Three studies had evidence of masking bias.

## DISCUSSION

To our knowledge, this network meta-analysis, including 5413 patients, is the first comprehensive study for assessing the efficacy and safety of ICI-related therapies for the treatment of advanced melanoma. In terms of PFS, ipilimumab plus nivolumab, nivolumab or pembrolizumab provided obvious clinical advantages over chemotherapy, ipilimumab, ipilimumab plus chemotherapy, ipilimumab plus sargramostim or ipilimumab plus gp100. For OS, pembrolizumab and nivolumab may be the most effective treatment strategies. As to safety, nivolumab or pembrolizumab induced less high-grade drug-related AEs than chemotherapy, ipilimumab, ipilimumab plus chemotherapy, ipilimumab plus nivolumab or ipilimumab plus gp100. Taken together, the analysis reveals that nivolumab or pembrolizumab is advantageous for the treatment of patients with advanced melanoma due to their balance between benefits and acceptability.

In this analysis, we found that the therapeutic antibodies targeting PD-1 could be superior to those targeting CTLA-4. Compared with chemotherapy, the first-line treatment for advanced melanoma [32], the anti-PD-1 antibodies including nivolumab and pembrolizumab significantly improved PFS and OS with lower incidence of high-grade AEs. However, the anti-CTLA-4 antibodies including ipilimumab and tremelimumab resulted in less satisfied outcomes. Compared with chemotherapy, ipilimumab failed to improve PFS and OS, and tremelimumab prolonged PFS not OS with increased high-grade AEs. The varied clinical profiles could be attributed to the functional characteristics of CTLA-4 and PD-1. CTLA-4 acts as a checkpoint for naive T-cells at the initial stage of immune response, while PD-1 only transduces inhibitory signals in activated T-cells at the late stage [33]. Thus, the CTLA-4 inhibition could lead to more T-cell clones against auto-antigens to be primed, and therefore induced higher frequency of high-grade AEs [33]. The correlation of CTLA-4 inhibition with the severe AEs was initially documented in CTLA-4 knock-out mice which developed fatal lymphocyte hyperproliferation [34]. The life-threatening side effects should disable the anti-CTLA-4 antibodies as more suitable therapeutic ICI targeting antibodies for the treatment of patients with advanced melanoma than anti-PD-1 antibodies. Considering its functional complementarity and nonredundancy, ipilimumab has been tested in combination with nivolumab for improving their anti-tumor efficacy. However, in this analysis, we failed to find synergic advantages of the combination on improving PFS, but found significant higher incidence of AEs. The HR for PFS of ipilimumab plus nivolumab versus nivolumab was close to 1, suggesting that combining ipilimumab with nivolumab is unnecessary for the treatment of advanced melanoma. Since this analysis was mainly based on summary statistics rather than patient-level longitudinal data, we couldn't adjust the combined efficacy with the expression of the PD-1 ligand (PD-L1) on tumor cells. PD-L1 expression might influence the efficacy of anti-PD-1 antibodies. In CheckMate 067 clinical trial, patients with PD-L1–negative tumors were found to have a higher rate of response and numerically longer PFS when treated with the combined therapy than with nivolumab alone, whereas patients with PD-L1–positive tumors had a similar PFS in two groups [12]. The results in this network meta-analysis together with the data from the original clinical trial suggest that the benefit with the combination of nivolumab and ipilimumab versus nivolumab alone may occur in the context of negative PD-L1 status. Thus, the use of PD-L1 as a biomarker may allow clinicians to make more informed decisions to combine ipilimumab with nivolumab or use nivolumab alone. However, caution is warranted in interpreting these data because the currently used method for assaying PD-L1 expression still needs to be improved and the efficacy of the combination therapy on OS is not yet known. The PFS provided by the existing clinical trials has less significance than OS for treatment selection, since measurement of PFS is less precise than that of OS, and might be affected by heterogeneity in follow-up across studies.

To fill a crucial knowledge gap regarding ICI-related therapies, we assessed the efficacy and acceptability of ipilimumab, ipilimumab plus sargramostim, ipilimumab plus chemotherapy, ipilimumab plus gp100 and ipilimumab plus budesonide simultaneously. Noticeably, this analysis showed that ipilimumab plus sargramostim was the most efficacious in improving OS among the combined therapies, and prolonged the OS by about a third compared to ipilimumab alone. The improvement was consistent with the data from the previous clinical trial [14]. Moreover, in this analysis, ipilimumab plus sargramostim even showed similar OS benefit when compared with the anti-PD-1 antibodies including nivolumab and pembrolizumab. The improved efficacy may be result from the synergy between sargramostim and ipilimumab. Sargramostim, a GM-CSF vaccine, could enhance the presentation of tumor-derived antigens via recruiting the macrophages and dentritic cells. Ipilimumab could suppress regulatory T cells (Tregs). The combination of GM-CSF-secreting tumor cell vaccines and anti-CTLA-4 antibody was also demonstrated to increase the ratio of tumor-infiltrating CD8^+^ cytotoxic T cells to Tregs in preclinical and clinical studies [35]. Interestingly, this analysis revealed that sargramostim could mitigate toxicity induced by ipilimumab. However, the addition of sargramostim failed to further improve the efficacy of ipilimumab on PFS. Such uncoupling of OS and PFS benefit was previously described in the treatment of advanced prostate cancer with sipuleucel-T [36]. The uncoupling may be attributed to the incompetent of conventional radiographic criteria to discern the inflammatory responses from tumor cells. Overall, the analysis on OS and safety suggests that besides the anti-PD-1 antibodies, ipilimumab plus sargramostim is also suitable for the treatment of advanced melanoma.

Among these combination therapies, ipilimumab plus gp100 and ipilimumab plus budesonide were the least efficacious. The non-effect of the gp100 might be due to the mutation of the gp100, which occurred in more than 90% of melanomas at various stages [36]. In addition, ipilimumab plus chemotherapy showed no further obvious survival benefit than ipilimumab alone, but significantly increased high-grade drug-related AEs. These results indicate an unnecessity to include gp100, budesonide or dacarbazine into the ipilimumab therapy for the treatment of advanced melanoma.

The strengths of our study are as follows. To the best of our knowledge, this study is the first meta-analysis to assess currently available ICI-related therapies for advanced melanoma. A comprehensive and rigorous search strategy was undertaken to retrieve all potentially eligible RCTs. In addition, we used Bayesian network meta-analysis to analyze the available data. This method incorporated all available high-quality randomized evidence regarding the efficacy of ICIs for advanced melanoma while fully maintaining randomization. We applied various statistical approaches to synthesize all available data and to increase reliability of the results. Consistent results were noted across all analyzed outcomes. Moreover, the reliability and accuracy of results were accompanied by the low statistical heterogeneity, absence of inconsistency, and excellent model fit. Finally, the combination of survival benefits and AEs could provide new insights into the benefit–risk ratio of different ICI-related therapies.

However, the limitations of this analysis need to be acknowledged. The main limitation of this network meta-analysis arises from the quality of the primary trials reviewed. Three included trials had definite evidences of masking bias, which might affect the validity of overall findings. Moreover, the analysis was performed based on summary statistics rather than patient-level longitudinal data. There might be some prognostic factors (*e.g.*, PD-L1 status, BRAF status, *etc*.) at the individual patient level that might influence the treatment efficacy, but were not available; therefore adjustment of these factors was impossible in the network meta-analysis. Access to data from individual patients could establish more robust conclusions in specific subgroups of patients. Finally, the length of follow-up and individual dosage varied across studies, which might somewhat limit the generalisability of our findings.

In conclusion, our network meta-analysis suggested that among the existing ICI-related treatments, the anti-PD-1 antibodies including nivolumab and pembrolizumab or ipilimumab plus sargramostim could be the optimal treatment for advanced melanoma. Anti-CTLA-4 antibodies, ipilimumab or tremelimumab, when administrated alone, could not provide obvious survival benefits. Ipilimumab plus nivolumab is the most effective in prolonging PFS but far more toxic than nivolumab, and therefore should be cautiously administrated.

## MATERIALS AND METHODS

### Literature-search strategy

A systematic literature search was conducted in Pubmed, Cochrane databases, Web of Science, and ClinicalTrials.gov for RCTs of ICIs for the treatment of advanced melanoma (Supplementary Materials for full search terms). The reference lists of included studies and related reviews were manually searched to identify additional trials. The search was performed in October 2016. There were no language or date restrictions.

### Inclusion and exclusion criteria

Studies were eligible if they satisfied the following criteria: (1) the trial enrolled patients who had histologically confirmed unresectable stage III or IV melanoma; (2) patients were randomly assigned to receive ICIs or in combination with other treatments. Patients could have received previous adjuvant therapy; (3) one or more of the outcomes of interest mentioned below were reported. Duplicates, secondary reporting of clinical trials and non-RCTs were excluded.

### Data extraction and quality assessment

Two investigators (Xin Li and Junpeng Wang) independently reviewed the full text of identified studies and extracted data into a structured data abstraction form, including baseline characteristics, inclusion and exclusion criteria, treatment strategies, and outcomes (PFS, OS, overall and high-grade (grade ≥ 3) drug-related AEs). We focused on high grade drug-related AEs because grade 1–2 AEs were inconsistently reported in identified studies and had lesser clinical significance. Corresponding authors were contacted if outcomes mentioned above were not adequately reported in the original article. The methodological quality of individual studies was appraised with the Cochrane risk of bias assessment tool [18]. Any discrepancies between researchers were resolved by consensus.

### Data synthesis and analysis

We initially did traditional pair-wise meta-analyses to compare the treatment strategies using Statav.12 (Stata Corp, College Station, TX, USA). We used the I^2^ statistic and the chi-square test to assess heterogeneity among studies. An I^2^ > 50% or a p value < 0.10 represented the existence of severe heterogeneity.

We then performed a Bayesian frame meta-analyses for all outcomes. For pooled analysis of PFS and OS, the reported HRs with 95% confidence intervals (CIs) were used as the summary statistic. For studies that did not report HRs, we estimated them using the technique described by Tierney *et al* [19]. For drug-related AEs, we calculated odds ratios (ORs) from the number of drug-related AEs for each treatment group of every trial. Both fixed-effects and random- effects models were conducted for the analyses [20].

Model fit was determined by the use of the deviance information criterion and between-study standard deviation [20, 21]. Convergence was assessed graphically according to the method described previously [22].

A key assumption behind network meta-analysis is that direct and indirect evidence on a specific comparison is consistency [22–24]. To explore inconsistency in the network, we compared results from the network meta-analysis with traditional pair-wise estimates [24].

To assess the robustness of the results from the primary analysis, we performed sensitivity analyses by restricting to trials with a low risk of bias. Funnel plots were used to evaluate small-study effects and publication bias.

We did the Bayesian network meta-analysis with Open BUGS version3.2.2 for PFS and OS, and GeMTC version 0.14.3 [25] for AEs, respectively. For PFS and OS, we used 30,000 iterations (10,000 per chain) obtained after a 10,000-iteration training phase. To minimize autocorrelation we used a thinning interval of 50 for each chain. For AEs, we computed ORs on averages of the 60,000 iterations, discarding the first 40,000 iterations.

## SUPPLEMENTARY MATERIALS FIGURES AND TABLES



## Abbreviations

ICI: immune checkpoint inhibitor
OS: overall survival
CTLA-4: cytotoxic T lymphocyte-associated antigen-4
PD-1: programmed death 1
AE: adverse event
ORR: overall response rate
PFS: progression-free survival
gp100: glycoprotein 100
GM-CSF: granulocyte-macrophage colony-stimulating factor
RCT: randomized controlled trial
HR: hazard ratio
CI: confidence interval
OR: odds ratio
PD-L1: PD-1 ligand
