# Supplementary material for: Comparative efficacy and safety of immune checkpoint inhibitor-related therapies for advanced melanoma: a Bayesian network analysis
Source: Oncotarget. 2017 Jul 1;8(48):83637–49. doi: 10.18632/oncotarget.18906 (PMC5663542; doi:10.18632/oncotarget.18906)
Supplement: Supplementary file 1 [file oncotarget-08-83637-s001.pdf]

Comparative efficacy and safety of immune checkpoint inhibitor-related therapies for advanced melanoma: a Bayesian network analysis

SUPPLEMENTARY MATERIALS

Search strategy

Pubmed:

("Melanoma"[Mesh]) AND ((((((((anti PD-L1[Title/Abstract]) OR aztezolizumab[Title/Abstract]) OR durvalumab[Title/Abstract]) OR avelumab[Title/Abstract]) OR BMS-986016[Title/Abstract])) OR ((((anti PD-1[Title/Abstract]) OR nivolumab[Title/Abstract]) OR pembrolizumab[Title/Abstract]) OR pidilizumab[Title/Abstract])) OR ((((anti CTLA4[Title/Abstract]) OR anti CTLA-4[Title/Abstract]) OR ipilimumab[Title/Abstract]) OR tremelimumab[Title/Abstract]))

Cochrane Library:

#1 anti PD-L1:ti,ab,kw or aztezolizumab or durvalumab or avelumab or BMS-986016 (Word variations have been searched)

#2 anti PD-1:ti,ab,kw or nivolumab:ti,ab,kw or pembrolizumab:ti,ab,kw or pidilizumab:ti,ab,kw (Word variations have been searched)

#3 anti CTLA4:ti,ab,kw or anti CTLA-4:ti,ab,kw or ipilimumab:ti,ab,kw or tremelimumab:ti,ab,kw (Word variations have been searched)

#4 MeSH descriptor: [Melanoma] explode all trees

#5 #1 or #2 or #3

#6 #4 and #5

Web of science

#1 Topic: (anti CTLA4) OR Topic: (anti CTLA-4) OR Topic: (ipilimumab) OR Topic: (tremelimumab)

#2 Topic: (anti PD-1) OR Topic: (nivolumab) OR Topic: (pembrolizumab) OR Topic: (pidilizumab)

#3 Topic: (anti PD-L1) OR Topic: (aztezolizumab) OR Topic: (durvalumab) OR Topic: (avelumab) OR Topic: (BMS-986016)

#4 Topic: (Melanoma)

#5 #1 OR #2 OR #3

#6 #5 AND #4

ClinicalTrials.gov:

Category: "Melanoma, Studies With Results"
(http://clinicaltrials.gov/)

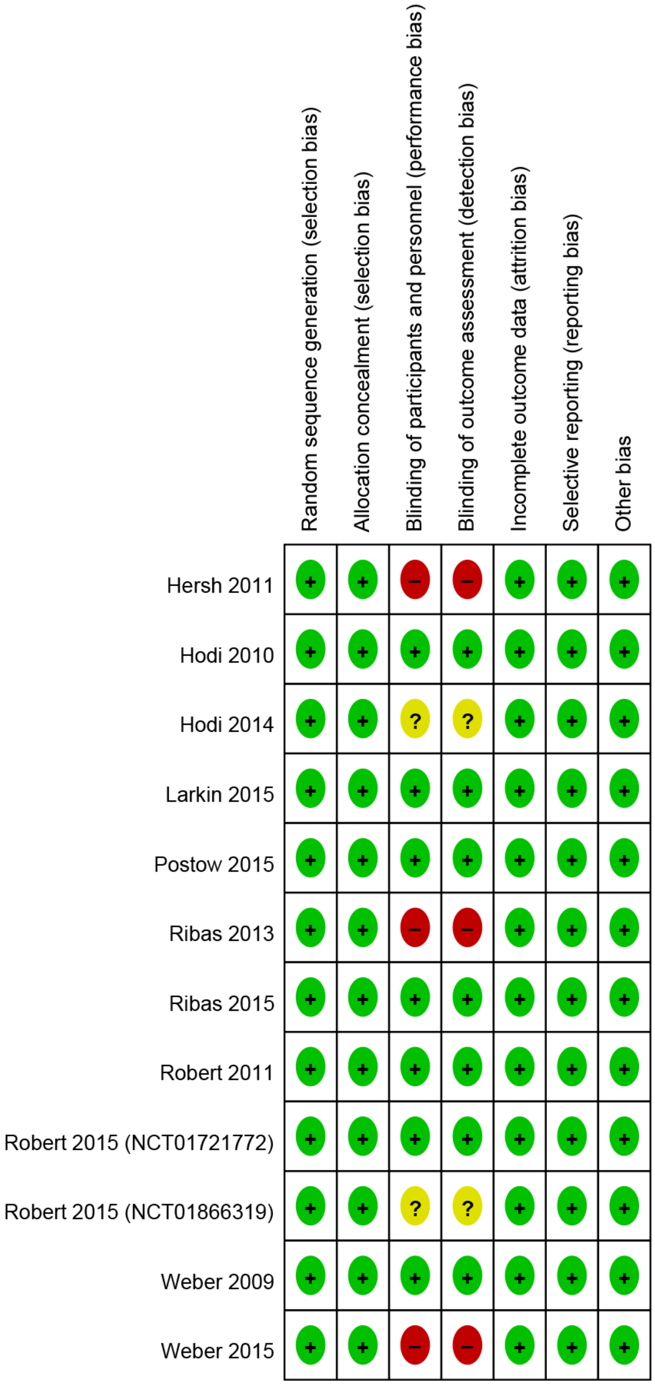

Supplementary Figure 1: Cochrane risk of bias tool assessment.

A

CHE	0.83 (0.48-1.41)	0.88 (0.75-1.04)	**0.42** (0.25-0.72)	**0.55** (0.31-0.96)	**0.72** (0.59-0.88)	0.86 (0.48-1.54)	0.53 (0.23-1.18)	0.86 (0.39-1.90)
	IPI	1.06 (0.61-1.87)	0.51 (0.24-1.09)	**0.66** (0.54-0.80)	0.87 (0.53-1.43)	1.04 (0.83-1.30)	0.64 (0.35-1.18)	1.04 (0.58-1.87)
		TRE	**0.48** (0.27-0.84)	0.62 (0.34-1.12)	0.82 (0.64-1.06)	0.98 (0.54-1.79)	0.60 (0.26-1.36)	0.98 (0.43-2.19)
			NIV	1.31 (0.60-2.85)	1.72 (0.97-3.04)	2.05 (0.93-4.53)	1.26 (0.48-3.32)	2.06 (0.78-5.34)
				PEM	1.32 (0.77-2.24)	**1.57** (1.17-2.12)	0.97 (0.51-1.83)	1.58 (0.85-2.93)
					IPI_ CHE	1.20 (0.69-2.07)	0.74 (0.33-1.60)	1.20 (0.55-2.57)
						IPI_GP 100	0.62 (0.32-1.17)	1.00 (0.53-1.87)
							IPI_ SAR	1.63 (0.69-3.81)
								IPI_ BUD

B

CHE	0.81 (0.36, 1.98)	2.31 (0.61, 8.46)	0.63 (0.29, 1.33)	0.77 (0.37, 1.68)	**3.71** (1.13, 14.46)	1.98 (0.59, 5.79)	1.64 (0.38, 7.57)	0.38 (0.04, 2.70)
	IPI	2.83 (0.55, 12.82)	0.77 (0.30, 1.76)	0.95 (0.44, 1.94)	**4.55** (1.31, 17.36)	2.44 (0.81, 5.85)	2.01 (0.58, 6.91)	0.46 (0.06, 2.69)
		TRE	0.27 (0.06, 1.23)	0.34 (0.08, 1.57)	1.63 (0.28, 11.12)	0.87 (0.15, 4.56)	0.71 (0.10, 5.46)	0.16 (0.01, 1.77)
			NIV	1.23 (0.50, 3.25)	**5.95** (1.62, 25.61)	**3.16** (1.01, 8.54)	2.60 (0.60, 12.62)	0.61 (0.07, 4.41)
				PEM	**4.81** (1.36, 19.88)	2.57 (0.76, 7.36)	2.10 (0.51, 9.08)	0.48 (0.06, 3.32)
					IPI_ CHE	0.53 (0.10, 2.16)	0.44 (0.07, 2.49)	0.10 (0.01, 0.86)
						IPI_NIV	0.82 (0.19, 4.37)	0.19 (0.02, 1.54)
							IPI_GP 100	0.23 (0.02, 1.93)
								IPI_ BUD

Supplementary Figure 2: Pooled hazard ratios for overall survival (A) and pooled odds ratios for overall adverse events (B) by Bayesian network meta-analysis. The column treatment is compared with the row treatment. For overall survival, HRs lower than 1 favour the column-defining treatment. For overall adverse events, ORs lower than 1 favour the column-defining treatment. Numbers in parentheses indicate 95% credible intervals. Significant results are in bold and underscored. CHE = chemotherapy. IPI = ipilimumab. TRE = tremelimumab. NIV = nivolumab. PEM = pembrolizumab. IPI_CHE = ipilimumab plus chemotherapy. IPI_NIV = ipilimumab followed by nivolumab. IPI_GP100 = ipilimumab plus glycoprotein 100. IPI_SAR = ipilimumab plus sargramostim. IPI_BUD = ipilimumab plus budesonide.

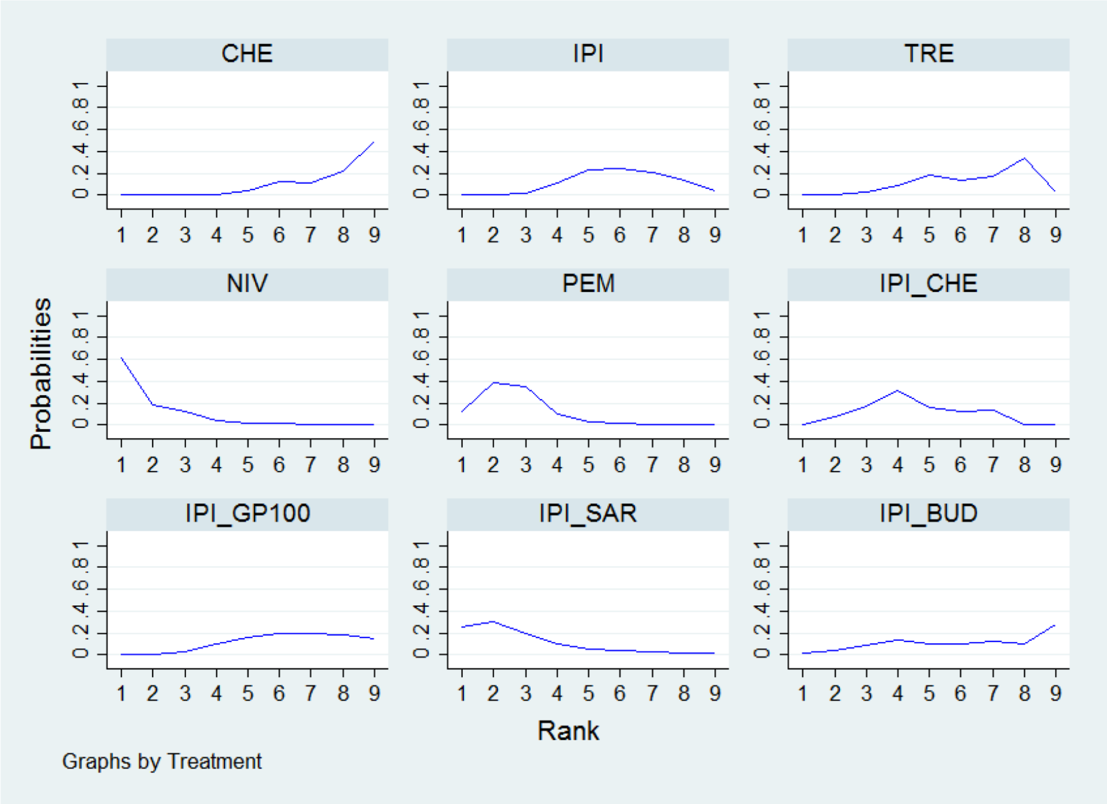

Supplementary Figure 3: Ranking of treatments in terms of overall survival benefit. Ranking indicates the probability to be the best treatment, the second best, the third best, and so on, among the 9 immune checkpoint inhibitor-related treatments. CHE = chemotherapy. IPI = ipilimumab. TRE = tremelimumab. NIV = nivolumab. PEM = pembrolizumab. IPI_CHE = ipilimumab plus chemotherapy. IPI_GP100 = ipilimumab plus glycoprotein 100. IPI_SAR = ipilimumab plus sargramostim. IPI_BUD = ipilimumab plus budesonide.

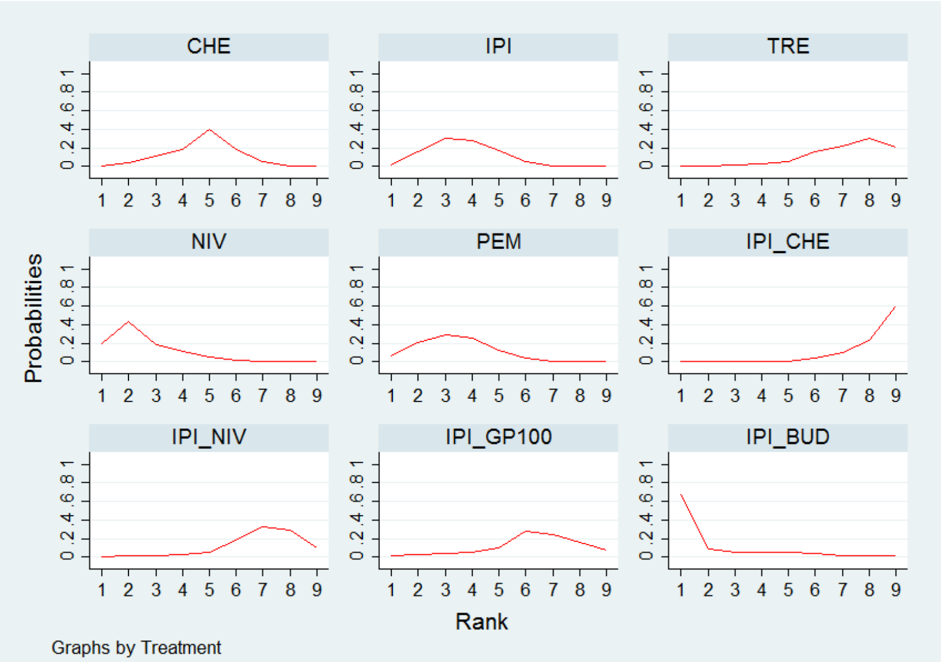

Supplementary Figure 4: Ranking of treatments in terms of overall adverse events. Ranking indicates the probability to be the best treatment, the second best, the third best, and so on, among the 9 immune checkpoint inhibitor-related treatments. CHE = chemotherapy. IPI = ipilimumab. TRE = tremelimumab. NIV = nivolumab. PEM = pembrolizumab. IPI_CHE = ipilimumab plus chemotherapy. IPI_NIV = ipilimumab followed by nivolumab. IPI_GP100 = ipilimumab plus glycoprotein 100. IPI_BUD = ipilimumab plus budesonide.

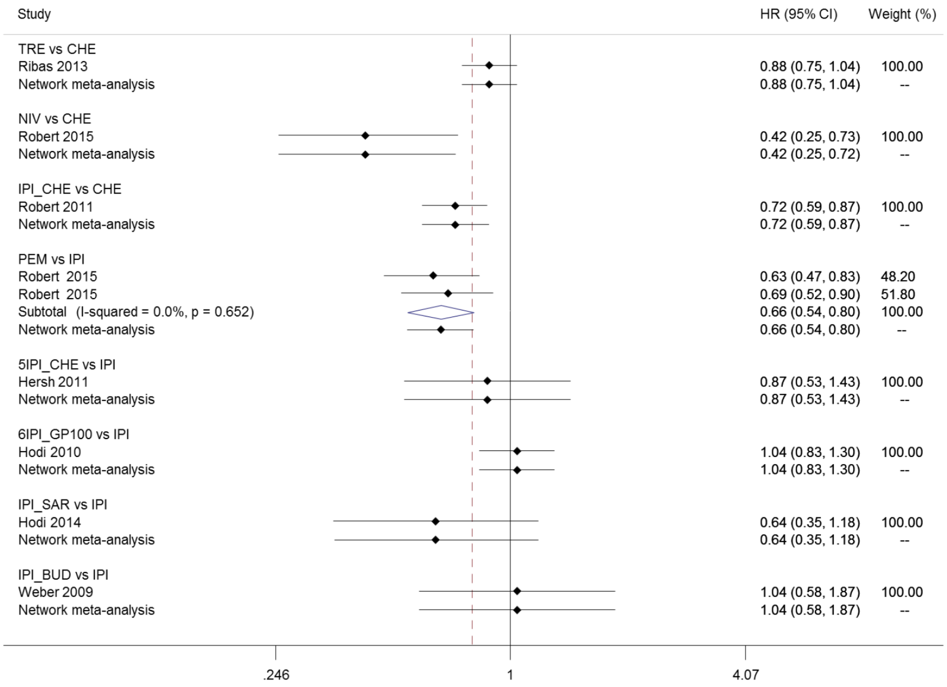

Supplementary Figure 5: Pooled hazard ratios for overall survival by Bayesian network-analysis and traditional meta-analysis. HR = hazard ratio. CI=confidence interval for traditional meta-analysis and credible interval for Bayesian network meta-analysis. CHE = chemotherapy. IPI = ipilimumab. TRE = tremelimumab. NIV = nivolumab. PEM = pembrolizumab. IPI_CHE = ipilimumab plus chemotherapy. IPI_GP100 = ipilimumab plus glycoprotein 100. IPI_BUD = ipilimumab plus budesonide. IPI_SAR = ipilimumab plus sargramostim.

Supplementary Table 1: High-grade adverse event- Evaluation of inconsistency by using nodesplit approach (all p-values >0.05)

Comparison	Direct Effect	Indirect Effect	Overall	P-Value
CHE vs NIV	-1.01 (-1.84, -0.18)	-0.78 (-2.16, 0.75)	-0.95 (-1.54, -0.33)	0.72
CHE vs PEM	-0.94 (-1.82, -0.07)	-0.77 (-2.16, 0.72)	-0.91 (-1.50, -0.28)	0.82
CHE vs IPI_CHE	1.24 (0.20, 2.24)	0.40 (-1.35, 2.18)	1.11 (0.22, 1.83)	0.37
IPI vs NIV	-0.66 (-1.74, 0.46)	-0.89 (-2.23, 0.36)	-0.72 (-1.43, -0.05)	0.71
IPI vs PEM	-0.63 (-1.47, 0.20)	-0.78 (-2.30, 0.57)	-0.68 (-1.29, -0.09)	0.82
IPI vs IPI_CHE	0.73 (-0.80, 2.27)	1.58 (0.33, 2.82)	1.34 (0.31, 2.14)	0.37
NIV vs IPI_NIV	1.87 (0.73, 2.96)	2.06 (0.58, 3.69)	1.90 (1.18, 2.74)	0.79

Numbers in parentheses indicate 95% credible intervals. CHE = chemotherapy. IPI = ipilimumab. TRE = tremelimumab. NIV = nivolumab. PEM = pembrolizumab. IPI_CHE = ipilimumab plus chemotherapy. IPI_GP100 = ipilimumab plus glycoprotein 100. IPI_BUD = ipilimumab plus budesonide. IPI_SAR = ipilimumab plus sargramostim.

Supplementary Table 2: Overall adverse event- Nodesplit approach for assessment of inconsistency (all p-values >0.05)

Comparison	Direct Effect	Indirect Effect	Overall	P-Value
CHE vs NIV	-0.33 (-1.31, 0.62)	-0.98 (-2.70, 0.83)	-0.47 (-1.24, 0.29)	0.4
CHE vs PEM	-0.52 (-1.39, 0.35)	0.38 (-0.93, 1.99)	-0.26 (-1.00, 0.52)	0.19
CHE vs IPI_CHE	1.75 (0.06, 3.73)	0.75 (-1.29, 2.82)	1.31 (0.12, 2.67)	0.42
IPI vs NIV	-0.37 (-1.62, 0.95)	0.15 (-1.50, 1.47)	-0.26 (-1.21, 0.56)	0.5
IPI vs PEM	0.18 (-0.67, 1.01)	-0.77 (-2.38, 0.64)	-0.05 (-0.83, 0.66)	0.18
IPI vs IPI_CHE	1.06 (-0.67, 2.90)	2.05 (0.10, 4.21)	1.52 (0.27, 2.85)	0.42
NIV vs IPI_NIV	1.51 (0.48, 2.65)	-0.26 (-2.18, 1.61)	1.15 (0.01, 2.14)	0.09

Numbers in parentheses indicate 95% credible intervals. CHE = chemotherapy. IPI = ipilimumab. TRE = tremelimumab. NIV = nivolumab. PEM = pembrolizumab. IPI_CHE = ipilimumab plus chemotherapy. IPI_NIV = ipilimumab followed by nivolumab.

Supplementary Table 3: Comparison of hazard ratios (95% CI) for progression-free survival from fixed and random models

Treatment compared with CHE	Fixed Model	Random Model
IPI	0.91 (0.73-1.14)	0.92(0.62-1.40)
TRE	**0.55 (0.39-0.77)**	**0.55(0.33-0.92)**
NIV	**0.45 (0.36-0.57)**	**0.47(0.34-0.74)**
PEM	**0.53 (0.45-0.63)**	**0.53(0.39-0.72)**
IPI_CHE	**0.76 (0.63-0.92)**	0.76(0.49-1.19)
IPI_NIV	**0.35 (0.24-0.50)**	**0.35(0.22-0.62)**
IPI_GP100	1.09 (0.83-1.42)	1.09(0.61-2.01)
IPI_SAR	0.80 (0.55-1.16)	0.80(0.43-1.53)
DIC	-5.75	-3.778

Numbers in parentheses indicate 95% credible intervals. CHE = chemotherapy. IPI = ipilimumab. TRE = tremelimumab. NIV = nivolumab. PEM = pembrolizumab. IPI_CHE = ipilimumab plus chemotherapy. IPI_NIV = ipilimumab followed by nivolumab. IPI_GP100 = ipilimumab plus glycoprotein 100. IPI_SAR = ipilimumab plus sargramostim. Bold type font indicates significant values.

Supplementary Table 4: Comparison of hazard ratios (95% CI) for overall survival from fixed and random models

Treatment compared with CHE	Fixed Model	Random Model
IPI	0.83 (0.48-1.41)	0.82 (0.0007-96.69)
TRE	0.88 (0.75-1.04)	0.88 (0.014-37.28)
NIV	**0.42 (0.25-0.72)**	0.42 (0.007-26.64)
PEM	**0.55 (0.31-0.96)**	0.54 (0.0002-107.8)
IPI_CHE	**0.72 (0.59-0.88)**	0.71 (0.006-24.58)
IPI_GP100	0.86 (0.48-1.54)	0.86 (0.0003-827.4)
IPI_BUD	0.86 (0.39-1.90)	0.85 (0.0004-448.9)
IPI_SAR	0.53 (0.23-1.18)	0.53 (0.0002-266.6)
DIC	0.9121	2.332

Numbers in parentheses indicate 95% credible intervals. CHE = chemotherapy. IPI = ipilimumab. TRE = tremelimumab. NIV = nivolumab. PEM = pembrolizumab. IPI_CHE = ipilimumab plus chemotherapy. IPI_NIV = ipilimumab followed by nivolumab. IPI_GP100 = ipilimumab plus glycoprotein 100. IPI_BUD = ipilimumab plus budesonide. IPI_SAR = ipilimumab plus sargramostim. Bold type font indicates significant values.

Supplementary Table 5: Comparison of odds ratios (95% CI) for high-grade adverse event from consistency and inconsistency models

Treatment compared with CHE	Consistency Model	Inconsistency Model
IPI	0.79 (0.42, 1.64)	0.78 (0.34, 1.84)
TRE	1.86 (0.75, 4.40)	1.86 (0.65, 5.20)
NIV	**0.39 (0.21, 0.72)**	**0.38 (0.19, 0.78)**
PEM	**0.40 (0.22, 0.75)**	**0.41 (0.20, 0.84)**
IPI_CHE	**3.04 (1.24, 6.22)**	**3.07 (1.12, 7.29)**
IPI_NIV	**2.62 (1.17, 6.51)**	2.49 (0.93, 7.44)
IPI_GP100	0.57 (0.19, 1.88)	0.55 (0.14, 2.16)
IPI_BUD	1.11 (0.32, 4.07)	1.08 (0.25, 4.91)
IPI_SAR	0.46 (0.14, 1.57)	0.46 (0.12, 1.90)
Random Effects Standard Deviation	0.27 (0.01, 0.91)	0.33 (0.04, 1.07)
Inconsistency Standard Deviation	NA	0.45 (0.02, 1.64)

Numbers in parentheses indicate 95% credible intervals. CHE = chemotherapy. IPI = ipilimumab. TRE = tremelimumab. NIV = nivolumab. PEM = pembrolizumab. IPI_CHE = ipilimumab plus chemotherapy. IPI_NIV = ipilimumab followed by nivolumab. IPI_GP100 = ipilimumab plus glycoprotein 100. IPI_BUD = ipilimumab plus budesonide. IPI_SAR = ipilimumab plus sargramostim. Bold type font indicates significant values.

Supplementary Table 6: Comparison of odds ratios (95% CI) for overall adverse event from consistency and inconsistency models

Treatment compared with CHE	Consistency Model	Inconsistency Model
IPI	0.81 (0.36, 1.98)	0.72 (0.31, 2.14)
TRE	2.31 (0.61, 8.46)	2.20 (0.62, 8.38)
NIV	0.63 (0.29, 1.33)	0.65 (0.27, 1.35)
PEM	0.77 (0.37, 1.68)	0.72 (0.35, 1.72)
IPI_CHE	**3.71 (1.13, 14.46)**	**4.10 (1.09, 17.24)**
IPI_NIV	1.98 (0.59, 5.79)	2.30 (0.57, 7.16)
IPI_GP100	1.64 (0.38, 7.57)	1.41 (0.35, 8.11)
IPI_BUD	0.38 (0.04, 2.70)	0.32 (0.04, 2.81)
Random Effects Standard Deviation	0.41 (0.06, 1.16)	0.38 (0.03, 1.20)
Inconsistency Standard Deviation	NA	0.74 (0.04, 1.47)

Numbers in parentheses indicate 95% credible intervals. CHE = chemotherapy. IPI = ipilimumab. TRE = tremelimumab. NIV = nivolumab. PEM = pembrolizumab. IPI_CHE = ipilimumab plus chemotherapy. IPI_NIV = ipilimumab followed by nivolumab. IPI_GP100 = ipilimumab plus glycoprotein 100. IPI_BUD = ipilimumab plus budesonide. Bold type font indicates significant values.

Supplementary Table 7: Comparison of results from primary analysis and sensitivityanalysis for trials with a low risk of bias

Treatment	Primary Analysis PFS HR (95% CI) vs CHE	Sensitivity Analysis PFS HR (95% CI) vs CHE
CHE	Reference	Reference
IPI	0.91 (0.73-1.14)	0.91 (0.73-1.13)
TRE	**0.55 (0.39-0.77)**	NA
NIV	**0.45 (0.36-0.57)**	**0.44 (0.35-0.55)**
PEM	**0.53 (0.45-0.63)**	**0.53 (0.45-0.63)**
IPI_CHE	**0.76 (0.63-0.92)**	**0.76 (0.63-0.93)**
IPI_NIV	**0.35 (0.24-0.50)**	**0.34 (0.23-0.49)**
IPI_GP100	1.09 (0.83-1.42)	1.08 (0.83-1.41)
IPI_SAR	0.80 (0.55-1.16)	0.79 (0.54-1.16)

HR = hazard ratio. CI = confidence interval. Numbers in parentheses indicate 95% credible intervals. CHE = chemotherapy. NA=data not available. IPI = ipilimumab. TRE = tremelimumab. NIV = nivolumab. PEM = pembrolizumab. IPI_CHE = ipilimumab plus chemotherapy. IPI_NIV = ipilimumab followed by nivolumab. IPI_GP100 = ipilimumab plus glycoprotein 100. IPI_SAR = ipilimumab plus sargramostim. Bold type font indicates significant values.
